# Rationale and design of a multi‐center, prospective randomized controlled trial on the effects of sacubitril–valsartan versus enalapril on left ventricular remodeling in ST‐elevation myocardial infarction: The PERI‐STEMI study

**DOI:** 10.1002/clc.23744

**Published:** 2021-10-20

**Authors:** Kaiyue Diao, Duolao Wang, Zhongxiu Chen, Xi Wu, Min Ma, Ye Zhu, Li Zhang, Hua Wang, Mian Wang, Sen He, Chen Li, Qiao Deng, Ting Yan, Tao Wu, Lu Tang, Baotao Huang, Jiayu Sun, Yong He

**Affiliations:** ^1^ Department of Radiology West China Hospital of Sichuan University Chengdu China; ^2^ Department of Clinical Sciences, Department of Biostatistics Liverpool School of Tropical Medicine Liverpool UK; ^3^ Department of Cardiology West China Hospital of Sichuan University Chengdu China; ^4^ Nursing Department West China School of Nursing, West China Hospital, Sichuan University Chengdu China

**Keywords:** angiotensin receptor neprilysin inhibitor, cardiovascular magnetic resonance, ST‐segment elevation myocardial infarction

## Abstract

**Background:**

Angiotensin receptor neprilysin inhibitor (ARNI) sacubitril‐valsartan has been recommended as one of the first‐line therapies in heart failure with reduced ejection fraction. However, whether ARNI could benefit patients with ST‐segment elevation myocardial infarction (STEMI) by improving left ventricular (LV) remodeling remains unknown. The primary objective of the PERI‐STEMI trial is to assess whether sacubitril‐valsartan is more effective in preventing adverse LV remodeling for patients with STEMI than enalapril.

**Hypothesis:**

We hypothesize that sacubitril/valsartan is superior to enalapril in preventing adverse LV remodeling evaluated by cardiovascular magnetic resonance imaging at the 6‐month follow‐up.

**Methods:**

PERI‐STEMI is an investigator‐initiated, prospective, multi‐center, randomized, open‐label, superiority trial with blinded evaluation of outcomes. A total of 376 first‐time STEMI patients with primary percutaneous coronary intervention (PPCI) within 12 h after symptom onset will be randomized to sacubitril‐valsartan or enalapril treatment. All the patients will receive a baseline cardiovascular magnetic resonance (CMR) examination at 4–7 days post‐PPCI. The primary endpoint is the change of indexed LV mass at the 6‐month follow‐up CMR.

**Results:**

Enrollment of the first patient is planned in November 2021. Recruitment is anticipated to last for 12–18 months and patients will be followed for 5 years after randomization. The study is expected to complete in June 2027.

**Conclusions:**

The results of the PERI‐STEMI trial are expected to provide CMR evidence on whether ARNI could benefit patients with STEMI, so as to facilitate the strategy of CMR‐based risk stratification and therapy selection for these patients. PERI‐STEMI is registered at ClinicalTrials.gov (NCT04912167).

## 1 BACKGROUND

Angiotensin receptor neprilysin inhibitor (ARNI) sacubitril‐valsartan, also known as LCZ696, has proven its superiority over enalapril in reducing adverse clinical events in patients with heart failure (HF) and reduced ejection fraction (HFrEF).^1^ Further study showed that treatment with sacubitril‐valsartan compared to valsartan alone is associated with significant improvement in cardiac function and reverse remodeling.^2^, ^3^ Although these benefits for HF with preserved ejection fraction (HFpEF) was not significant, a number of studies have reported that use of sacubitril‐valsartan could result in a lower level of N‐terminal pro‐B‐type natriuretic peptide (NT‐proBNP) and an improvement in left atrial function.^4^, ^5^, ^6^ Given that HF represents a heterogenous group of patients, and a differential treatment effect in this broad population raised by previous data, studies on specific subtype of patients with HFrEF or HFpEF are warranted to recognize the target patients who might benefit most from sacubitril‐valsartan treatment.^5^

Ischemia heart disease (IHD), especially a history of myocardial infarction (MI), is a common etiology of HF.^7^, ^8^ Such patients harbor a high risk of recurrent cardiovascular events and poor prognosis.^9^, ^10^ Early presence and pattern of left ventricular (LV) remodeling were closely associated with the long‐term outcome and should be carefully evaluated.^11^, ^12^, ^13^ Angiotensin‐converting enzyme inhibitors (ACEI) or angiotensin receptor blocker (ARB) are thus suggested for consideration in all MI patients, owing to their additional benefits and protecting mechanism on the heart and vasculature remodeling.^14^, ^15^, ^16^ Sacubitril‐valsartan is efficacious in preventing maladaptive cardiac fibrosis and prevent adverse LV remodeling compared to ACEI treatment.^17^ This superiority has been proven in hypertension, acute HF and MI animal models.^18^, ^19^ Nevertheless, whether it is effective in preventing adverse remodeling, or improving reverse remodeling among patients with ST‐segment elevation MI (STEMI) remains unknown.

Thus, we present the PERI‐STEMI (*P*rospective comparison of *E*arly *R*emodeling *I*maging between ARNI sacubitril/valsartan and ACEI enalapril in *ST*‐*e*levation *m*yocardial *i*nfarction patients) trial. The primary objective of this study is to decide whether sacubitril‐valsartan is more effective in preventing adverse LV remodeling for patients with STEMI, compared to enalapril. We hypothesize that sacubitril/valsartan is superior to enalapril in preventing adverse LV remodeling evaluated by cardiovascular magnetic resonance (CMR) imaging at the 6‐month follow‐up. Secondary objectives include: (a) To examine the long‐term outcome of sacubitril‐valsartan in STEMI, as compared to enalapril; and (b) to explore the capacity of baseline CMR markers in predicting patients who might benefit from treatment of sacubitril–valsartan.

## 2 METHODS

### 2.1 Study design

**FIGURE 1:**
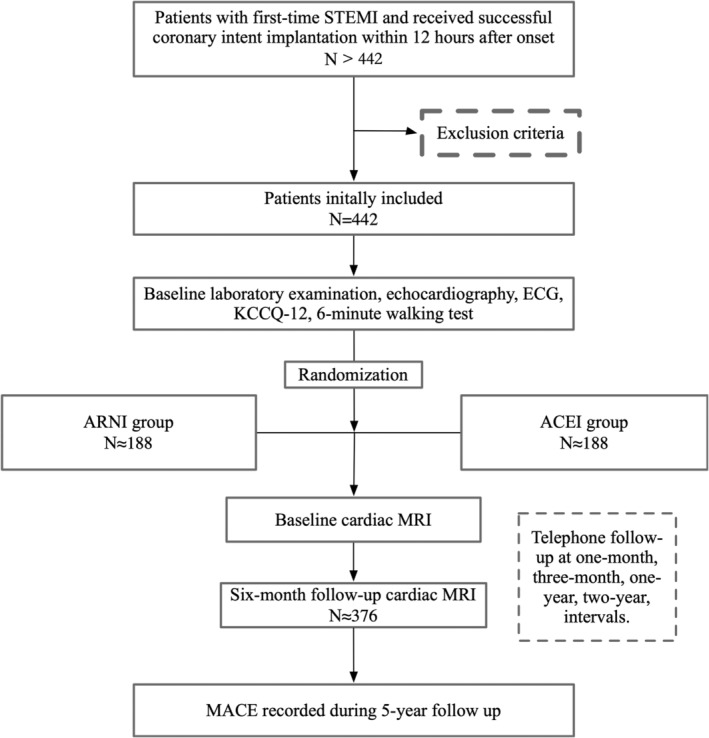
Study plan. ACEI, angiotensin‐converting enzyme inhibitors; ARNI, angiotensin receptor neprilysin inhibitor; ECG, electrocardiogram; MACE, major adverse, cardiac event; MRI, magnetic resonance imaging; STEMI, ST‐segment elevation myocardial infarction

This study is an investigator‐initiated, multi‐center, open‐label, parallel‐group, superiority study (Figure 1). Patients will be recruited from approximately 20 hospitals in China. This study has been approved by the ethic committee of West China hospital and all participating centers. PERI‐STEMI is registered at ClinicalTrials.gov (NCT04912167).

### 2.2 Patient eligibility

Detailed inclusion and exclusion criteria are presented in Table 1. Briefly, continuous patients aged between 18 and 75 and referred to the designated chest pain center with the diagnosis of spontaneous STEMI will be initially screened. The diagnosis of STEMI will be decided by the physician based on the newest clinical guidelines.^14^, ^20^ Considering that multiple factors may affect ventricular remodeling, this study will only include patients with a uniform baseline condition, namely first‐onset STEMI, successfully treated by primary percutaneous coronary intervention (PPCI) within 12 h after onset, and hemodynamically stable to be eligible for further evaluation. Hemodynamically stable is defined as fulfilling the following criteria: (a) systolic blood pressure (SBP) ≥ 100 mmHg for patients who received ACEI/ARB during the last 24 h or SBP ≥110 mmHg for patients who did not, (b) no increase intravenous treatment with diuretics or vasodilators, and (c) no vasopressors and/or inotropes during the last 24 h prior to randomization.^21^

**TABLE 1:** List of inclusion and exclusion criteria for patients

List of inclusion and exclusion criteria for patients
*Inclusion criteria*
Aged between 18 and 75 years old
First‐time ST‐segment elevation myocardial infarction based on the newest ESC guidelines^a^
Timely primary percutaneous coronary intervention within 12 h from onset^b^
Written informed consent acquired
*Exclusion criteria*
Patients with symptomatic hypotension, or a systematic blood pressure less than 100 mm Hg at screening or 95 mm Hg at randomization.
Patients with Takotsubo cardiomyopathy or myocardial infarction secondary to another medical condition such as anemia, hypotension, or arrhythmia, coronary vasospasm
Known history of or persistent clinical chronic heart failure prior to randomization
Previous use of ARNI, or intolerance or contraindications to study drugs including ARNI or ACEI
History of significant chronic coronary obstruction and adverse ventricular remodeling
History of any cardiomyopathy, valvular heart disease, congenital heart disease, stent or CABG, or planned open‐heart surgery within 3 months
History of hepatic impairment or history of cirrhosis with evidence of portal hypertension
History of chronic renal dysfunction, or eGFR <30 ml/min/1.73 m^2^
History of malignancy and with a life span less than 1 year
Patients with a known history of angioedema related to previous ACEIs/ARB therapy.
With contraindication to MRI examination (pacemaker and claustrophobia) or cannot finish breath‐holding when lying on the examination bed
Pregnancy or nursing women

Abbreviations: ACEI, angiotensin‐converting enzyme; ARB, angiotensin‐receptor blocker; ARNI, angiotensin receptor neprilysin inhibitor; CABG, coronary artery bypass graft; eGFR, estimated glomerular filtration rate; ESC, European Society of Cardiology; MRI, magnetic resonance imaging.

^a^ Including: (a) evidence of myocardial injury (defined as an elevation of cardiac troponin values with at least one value above the 99th percentile upper reference limit) with necrosis in a clinical setting consistent with myocardial ischemia; and (b) ST‐segment elevation in at least two contiguous leads.

^b^ The definition of successful revascularization requires (a) a minimum stenosis diameter reduction to 20% for the culprit vessel; (b) a grade 3 TIMI flow assessed by angiography.

### 2.3 Randomization and binding procedure

No run‐in period is included for PERI‐STEMI. Following screening patients who fulfill the eligibility criteria will be randomized (1:1) to either sacubitril–valsartan or enalapril. Permuted block randomization stratified by center will be employed. Randomization will be performed through an Interactive Web‐based Response System, for which a random allocation sequence is created using a standard computerized random‐number generator. This study is an open‐label trial, but the radiologists responsible for imaging analysis will be rigorously blind to the clinical and allocation information.

### 2.4 Treatment protocol

Standard peri‐PPCI clinical management will be applied for each participant. The prescriptions of anticoagulants, glycoprotein IIb/IIIa inhibitors, and thrombus aspiration will be left to the physician's discretion. For the patients with multivessel disease (MVD), revascularization of the non‐ischemia related artery is suggested but whether to perform this immediately or in a staged way will be left to the physician's discretion. Subsequently, patients randomized to ARNI group will receive two doses of ARB to ensure a minimum 36‐h washout period prior to initiation of ARNI therapy, and then be started with the first dose or sacubitril‐valsartan, while patients randomized to ACEI group will directly start with the first dose of enalapril. All patients will be monitored for hypotension 6 h after study treatment. In accordance with the current clinical management guidelines, all patients will receive a dual‐antiplatelet drug (aspirin, clopidogrel, or ticagrelor), β‐blocker agent and statins as the standard of post‐STEMI care. The use of sodium‐dependent glucose cotransporter 2 inhibitor (SGLT‐2i) for patients with diabetes will be left to the physician's discretion and recorded.

After initiation of treatment with sacubitril‐valsartan or enalapril, dose of the treatment will be titrated to a target level based on systolic blood pressure of the patients (Table 2). Clinicians are encouraged to uptitrate sacubitril‐valsartan and enalapril to target dose.

**TABLE 2:** Target treatment dose levels based on systolic blood pressure

Dose level	Sacubitril‐valsartan	Enalapril	Systolic blood pressure (SBP)
1	24/26 mg bid	2.5 mg bid	Initial SBP within 100–120 mm Hg and reducing to <100 mg Hg post‐treatment
2	49/51 mg bid	5 mg bid	Initial SBP within 100–120 mm Hg and maintaining ≥110 mm Hg post‐treatment, or Initial SBP ≥120 mm Hg but reducing to <100 mg Hg post‐treatment
3	97/103 mg bid	10 mg bid	Initial SBP ≥120 mm Hg and maintaining ≥110 mm Hg post‐treatment

*Note*: Bid: twice a day.

### 2.5 Imaging study and analysis

Image acquisition will be performed twice by qualified cardiac MRI experts with over 5 years working experience of cardiac MR imaging. The participating centers need to be equipped with MRI scan on 1.5 T or 3.0 T platforms with dedicated cardiac receiver coils. During imaging, the patients will be kept in supine position. A standard electrocardiographic triggering device will be used for heart rate triggering and monitoring.

**FIGURE 2:**
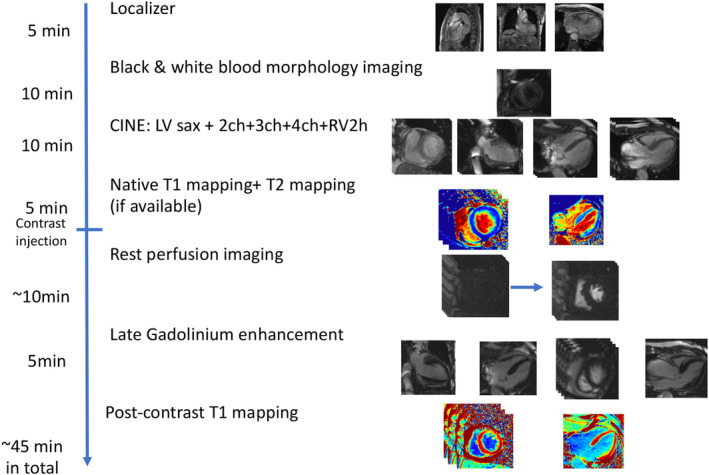
Cardiovascular magnetic resonance imaging protocol. 2ch, two chamber, 3ch, three chamber, 4ch, four chamber, LV, left ventricle, RV, right ventricle

A sample protocol is provided for all modalities (Figure 2). The participating sites will be allowed to use their own acquisition protocols, provided that standard cine, late gadolinium enhancement (LGE) and mapping images should be acquired according to the current practice guidelines. In the sample protocol, a balanced steady state free precession sequence (bSSFP) will be used to acquire continuous short‐axis (SAX) slices encompassing the whole LV, standard two‐, three‐, and four‐chamber cine images during repeated breath holds. After then, a dose of 0.1 mmol/kg Gadolinium will be injected at a flow rate of 2.5–3.0 ml/s. LGE images will be acquired 10–15 min after contrast administration using a phase‐sensitive inversion recovery sequence. Pre‐contrast T1 mapping and T2 mapping will be acquired, and a repeat MOLLI T1 mapping will be performed after LGE for the post‐T1 mapping.

All the image data will be sent to a core‐laboratory located at a dedicated high‐volume and experienced hospital for analysis. Two analysts with at least 5 years working experience in CMR analysis will perform the image analysis independently and blindly to the clinical data or treatment allocation information. Any discrepancy between the two radiologists will be referred to a superior radiologist and cardiologist to adjudicate.

### 2.6 Baseline and follow‐up examinations

All patients will be followed for a period of 5 years after PCI (including an in‐patient visit at 6 months, one required out‐patient visit at 30 days and 1 year, and seven another out‐patient or telephone visits). Echocardiography, electrocardiogram, 6‐min walk test and laboratory examinations will be taken at baseline, 6‐month in‐patient visit, and 1‐year out‐patient visit. CMR examinations and life quality evaluations using the Kansas City Cardiomyopathy Questionnaire (KCCQ‐12) will be performed at the acute phase (4–7 days after PCI) and chronic phase (6 months after PCI) respectively. For each visit, patients will be assessed for major adverse cardiac events (MACE) and any medical treatments will be recorded (details in Supporting information A).

### 2.7 Clinical data collection and monitoring

All study data will be recorded in a secure electronic data capture system (EDC) designed based on a case record form (CRF) that enables logging of all data entries. For each patient, only the subject number and initials will be recorded in the CRFs. The demographic data, laboratory examination results, PCI procedure records, ECG and echocardiography records will be obtained for each patient by a trained clinical research coordinator. The coordinator will maintain a personal subject identification list to make sure that all records are identified. All investigators will have access to the EDC. The study is monitored by an independent Data and Safety Monitoring Board (DSMB), which will be monitoring the safety of the participants and the interim efficacy analysis. Additionally, an independent Central Endpoint Committee (CEC) will be set up to adjudicate the deaths and important clinical outcomes. The CEC will be rigorously blinded to group allocation of the patients. (Details in Supporting information B).

### 2.8 Endpoints definition

The main endpoint is selected to characterize the effect of ARNI on ventricular remodeling as compared to ACEI. Based on the results from previous studies on LV remodeling, change of the indexed LV mass (Δ LVmassi) from baseline to 6‐month follow‐up on CMR is set to be the measurement for the primary outcome.^22^

Similarly, other secondary CMR outcomes include other remodeling parameters from baseline to 6‐month follow‐up (Δ left ventricular ejection fraction [LVEF], Δ LVEDV, Δ LVESV), peak LV strains (global radial peak strain, global circumferential peak strain, and global longitudinal peak strain), strain rates (global radial peak strain rate, global circumferential peak strain rate, and global longitudinal peak strain rate), infarction size as measured through LGE, presence of microvascular obstruction (MVO) on LGE, presence of iron load evaluated on T2* images, T1 mapping, and T2 mapping indexes at the 6‐month CMR.

### 2.9 The long‐term effects of ARNI on LV remodeling and clinical outcomes in patients will also be assessed in our study

Thus, key laboratory biomarkers are examined through both arms from baseline to 5‐year follow‐up: (a) HF related laboratory markers (NT‐proBNP, soluble growth stimulating express gene 2 (sST2); (b) myocardium injury related laboratory markers (cardiac troponin T, interleukin [IL]‐1, IL‐6, etc.); (c) MACE, including all deaths (cardiac death vs. non‐cardiac death^23^), non‐fatal myocardial re‐infarction,^24^ hospitalization for worsening heart failure or need for advanced HF therapies (hospital stay >24 h)^25^ (e.g., intravenous use of inotropes, LV assist device placement, or cardiac transplantation) (details in Supporting information C).

The safety assessments for this study include adverse events as follows: incidence of angioedema, symptomatic hypotension, renal insufficiency (i.e., assessed by serum creatinine, estimated glomerular filtration rate [eGFR] and presence of proteinuria, hematuria, and glycosuria), and hyperkalemia. The incidence of the above adverse events will be reported (Supporting information D).

No prespecified sub‐studies are planned in this study.

### 2.10 Sample size calculation

The sample size is estimated based on the primary endpoint measurement, that is, Δ LVmassi. Based on the review of published studies and a meta‐analysis summarizing the reported LV remodeling indexes between ARNI group and ACEI group, an average of approximately 4.81 g/m^2^ should be expected for the difference in Δ LVmassi at 6 months from baseline between the ARNI group and ACEI group, with an SD to be 16.65 g/m^2^.^12^ A sample size of 188 patients for each group (i.e., a total of 376 patients) was chosen to achieve ≥80% power for testing superiority of the ARNI group. Considering a maximum of 15% loss to follow‐up (based on the reported follow‐up rate at 4.3%–20%), a target sample size of 442 patients are planned to be initially recruited.^26^, ^27^

### 2.11 Statistical analysis

Primary trial analyses will be based on the modified intention to treat (ITT) population in which patients without a measurement of primary outcome will be excluded and additional analyses will also be performed on the per protocol population (PP). Considering that patients who received implantation of implantable cardioverter defibrillator (ICD) or cardiac resynchronization therapy (CRT) within 6 months will be unable to receive the second MRI examination, ITT population will consist of all randomized patients with valid outcome measurement. PP population is a subset of the ITT population in which patients with major protocol deviations will be excluded. Protocol deviations will be defined in the statistical analysis plan.

For the analysis of the primary outcome (change of the indexed LV mass (Δ LVmassi) from baseline to 6‐month follow‐up on CMR), a linear regression model will be employed with treatment as the study variable and baseline measurement of indexed LV mass as a covariate. In addition, adjusted linear model analysis will be performed with the pre‐specified covariates (LVEF, anterior infarction, MVD, presence of MVO on LGE, diabetes, hypertension, and the use of SGLT2i) measured at baseline being added into the above linear model. The crude and adjusted mean differences in the primary outcome together with its 95% confidence intervals at 6‐months will be derived from the linear models. In addition, subgroup analysis of primary endpoint will be performed on the above pre‐specified covariates.

Analysis of secondary continuous outcomes with single follow‐up measurement will be done in a similar fashion as the primary endpoint analysis. Analysis of secondary continuous outcomes with repeated follow‐up measurement will be performed using a linear mixed model with treatment visit, interaction between treatment and visit as fixed effects, the baseline value of the outcome as covariate if it is available, and subject as random effects. The analysis of binary outcomes will also use a generalized linear/mixed model depending on whether there will be a repeated measurement. Odds ratios with their 95% confidence intervals will be derived from the generalized linear/mixed model analysis. For the analysis of time‐to‐event outcome, Kaplan–Meier curves will be presented and compared by log rank test by treatment group, and hazard ratio and its 95% CI will be calculated using Cox regression model with the treatment arm as the study variable.

Missing primary outcome and secondary outcomes with a single measurement will not be imputed but missing secondary outcomes with repeated measurements will be imputed in sensitivity analysis using the last observation carried forward strategy. Missing baseline covariates will be imputed using simple imputation methods in the covariate adjusted analysis based on the covariate distributions in the sample. For a continuous variable, missing values will be imputed from random values from a normal distribution with mean and SD calculated from the available sample. For a categorical variable, missing values will be imputed from random values from a uniform distribution with probabilities *P*_*1*_, *P*_*2*_,…, and *P*_*k*_ from the sample. Seed for the imputation is set as the date of data analysis (e.g., 270 521).

The study is powered to make a single comparison in the primary outcome at 6 months only and other comparisons are exploratory in nature. Therefore, there is no multiplicity issue. All analyses will be described in detail in the statistical analysis plan. SAS 9.4 will be employed for the statistical analyses.

## 3 RESULTS

The study is estimated to be started in November 2021. Recruitment is supposed to be finished by the end of 2022. Primary results of the trial are anticipated in June 2023. The planned end date for the trial is June 2027.

## 4 DISCUSSION

The major purpose of PERI‐STEMI trial is to decide whether ARNI could bring superior cardiovascular benefit for patients with STEMI, when it is compared to traditional ACEI treatment. Additionally, through meticulous morphology, function and tissue characterization analysis on CMR, we also aimed to investigate the mechanism of ARNI in improving reverse remodeling, as well as seek CMR indexes for predicting maximum benefit from ARNI among such patients. Potentially, we could provide imaging evidence on application of ARNI in STEMI, and help elucidate the cardiovascular protection mechanism of this medicine.

The most innovative design of this trial is utilization of CMR for the evaluation of ventricular remodeling, cardiac function improvement, and other changes in the comparison between the patients. The promising cardiovascular effects of ARNI for patients with high LV load or who are prone to heart failure have been proven in previous studies. Several animal studies also produce positive evidence on implementation of ARNI in MI.^3^, ^28^ A recent study by Rezq et al demonstrated that compared to ACEI, ARNI is superior in preventing early (at 30 days) adverse clinical events and improving reverse remodeling for patients with STEMI by using echocardiography.^29^ Furthermore, a recent meta‐analysis on LV remodeling studies comparing ARNI versus ACEI/ARB in a heterogenous group of patients reported a distinct improved LV size and hypertrophy for patients prescribed with ARNI, even after short‐term follow‐up (shortest at 3 months in patients with essential hypertension).^22^ Especially, LVmassi presents significant difference in patients with both reduced and preserved LVEF. Nevertheless, CMR study in this scenario is rare. Considering that CMR is recommended as gold standard in cardiac function measurements, our trial sets the CMR‐derived 6‐month remodeling indexes as the primary outcome measurement.^30^, ^31^

Apart from accurate morphological and functional assessment, CMR's incremental prognostic value in predicting long‐term mortality and non‐vital cardiovascular events was also recognized, due to its unique capacity to observe and quantify myocardial fibrosis progression, edema and other tissue characterization change through LGE and mapping sequence.^32^, ^33^ Myocardial stunning is quite commonly seen in patients with STEMI and CMR, especially, and has unique ability to assess myocardial viability by providing accurate quantification of scar burden and myocardium perfusion.^34^, ^35^ Whether ARNI could provide cardiovascular benefits through improving myocardial viability and whether ARNI has a different role in patients with and without myocardium stunning warrant further investigation. A recent study suggested use of CMR for further risk stratification on STEMI patients with lower LVEF on echocardiography.^36^ Thus, with a multiple‐sequence design in our CMR protocol for this trial, we proposed that PERI‐STEMI could better demonstrate the advanced functional benefits ARNI could bring, as well as pave understanding of its cardiovascular mechanism.

Beyond comparing the function on early ventricular remodeling between ARNI and ACEI, another major clinical implication of this study will be that whether ARNI could bring long‐term benefits for patients with STEMI. Another published trial on the use of ARNI in acute MI, namely PARADISE‐MI trial, only included patients with an LVEF ≤40%. And their newly reported results indicated numerical but not statistical difference between ARNI group and ACEI group regarding the pre‐specified primary outcome (https://accscientificsession.acc.org). In addition, the results from PARADISE‐MI trial indicated that long‐term use of ARNI might help improve the outcome. Thus, more evidence is warranted in this field. However, LVEF is only a crude estimate of LV function.^37^ It is not sensitive to myocardial injury, or an ideal predictor for developing adverse LV remodeling.^38^, ^39^ A certain number of patients with normal LVEF developed adverse clinical outcomes while some patients with reduced LVEF at acute phase improved in the long‐term follow‐up.^40^, ^41^ Vaduganathan et al's study provided evidence that an LVEF of 40%–55% might also benefit from several HF treatments, suggesting a potential benefit of ARNI in part of the patients with preserved LVEF, especially for STEMI who are already at high risk of poor prognosis.^42^, ^43^ Thus, it might be inappropriate to arbitrarily only include patients with reduced LVEF when assessing the efficacy of ARNI in STEMI. Considering that no standard cut‐off for other CMR indexes has been set up to decide the patients at stake, our trial is designed not to exclude patients only based on imaging markers immediately after PPCI. Hopefully, this could contribute by answering the question whether additional CMR indexes could be used for further risk stratification of the patients with STEMI.

Another merit of this trial is that we rigorously include patients with timely revascularization, and who are hemodynamically stable. Considering the potential effects PPCI procedure might have on the baseline myocardium characterization of the patients, inclusion criteria is enriched to avoid the bias from procedure or the baseline condition of the patients. In addition, for these patients, possibility of having a CRT is relatively low, and thus a later exclusion could be prevented.

### 4.1 Limitations

As with any other clinical trial, our study has several limitations that should be mentioned. First, only hemodynamic stable patients who receive successful revascularization within 12 h, and who do not receive CRT or ICD implantation within 6 months will be finally included, thus certain bias exists and the interpretation of our results should be careful. Second, there might be a regional variation in the clinical outcomes in this multi‐center study. The study is powered to assess the difference in the change of the indexed LV mass (Δ LVmassi) from baseline to 6‐month follow‐up on CMR, which is a surrogate endpoint. We expect to collect some data on clinical outcomes from this study to plan a future large randomized clinical trial to assess the clinical efficacy of ARNI. Third, the major index observed in this study is an imaging marker, which will be measured objectively through an independent and blinded Core Laboratory. However, bias might still exist because the patients are not blinded. Nevertheless, LV remodeling is a relatively objective change and has not been reported related to the patient's subjective feeling. Measurements of primary and secondary outcomes including both imaging reading and clinical events assessment, data management and development of statistical analysis plan will be kept rigorously blinded to avoid potential bias. Finally, as this is an MRI study and intended to compare the effects of ARNI versus ACEI on LV remodeling, the results of the study will need to be carefully interpreted when transferring to clinical application. Although clinical outcomes will also be assessed in our study, those analyses will be exploratory in nature as the study is not powered to assess the effects of ARNI on clinical outcomes.

## 5 CONCLUSION

Patients with STEMI are prone to heart failure and adverse ventricular remodeling is a risk factor at early follow‐up. The PERI‐STEMI trial will be the first CMR study to test the effects of ARNI in patients with STEMI. Results of this trial will provide evidence on implementation of ARNI therapy in STEMI, and help elucidate the cardiovascular protection mechanism of ARNI.

## Supporting information


**Appendix** **S1**: Supporting Information.

## Data Availability

Data sharing not applicable to this article as no datasets were generated or analysed during the current study.
