# Supplementary material for: Rationale and design of a multi‐center, prospective randomized controlled trial on the effects of sacubitril–valsartan versus enalapril on left ventricular remodeling in ST‐elevation myocardial infarction: The PERI‐STEMI study
Source: Clin Cardiol. 2021 Oct 20;44(12):1709–17. doi: 10.1002/clc.23744 (PMC8715395; doi:10.1002/clc.23744)
Supplement: Supplementary file 1 — Appendix S1: Supporting Information. [file CLC-44-1709-s001.docx]

**Appendix**

**Table of Contents**

**Sup A. Planned follow-up visits and contents**

**Sup B. Study committees**

**Sup C. Clinical endpoint definitions**

**Sup D. Safety endpoint definitions**

**Sup A. Planned follow-up visits and contents**

| **Contents** | **Follow-up timeline** | | | | | | | | |
| --- | --- | --- | --- | --- | --- | --- | --- | --- | --- |
|  | **30 days** | **90 days** | **180 days** | **270 days** | **1 year** | **2 years** | **3 years** | **4 years** | **5 years** |
|  | Visit 1 | Visit 2 | Visit 3 | Visit 4 | Visit 5 | Visit 6 | Visit 7 | Visit 8 | Visit 9 |
| **In-patient visit** |  |  | **√** |  |  |  |  |  |  |
| **Out-patient visit** |  | **√** |  |  | **√** |  |  |  |  |
| **Telephone/out-patient visit** | **√** |  |  | **√** |  | **√** | **√** | **√** | **√** |
| **Medicine Taking** | **√** | **√** | **√** | **√** | **√** | **√** | **√** | **√** | **√** |
| **Laboratory examinations** |  |  | **√** |  | **√** |  |  |  |  |
| **Electrogram** | **√** |  | **√** |  | **√** |  |  |  |  |
| **Echocardiography** |  |  | **√** |  | **√** |  |  |  |  |
| **CMR** |  |  | **√** |  |  |  |  |  |  |
| **KCCQ-12** |  |  | **√** |  |  |  |  |  |  |
| **6-minute walk** |  |  | **√** |  | **√** |  |  |  |  |
| **MACE** | **√** | **√** | **√** | **√** | **√** | **√** | **√** | **√** | **√** |

CMR, cardiovascular magnetic resonance, KCCQ-12, Kansas City Cardiomyopathy Questionnaire, MACE, major adverse clinical events

**Sup B. Study committees**

**Executive Committee**

The Executive Committee is composed of Yong He and Jiayu Sun (Co-chairmen), Duolao Wang (Senior statistician, UK), Kai-yue Diao, Kang-hua Ma and Jing Wu. It provides scientific direction of the study and assesses the study progress. The Executive Committee will meet periodically and be assisted by the Steering Committee. The Executive Committee Chairmen are responsible for communicating with the DSMB and sponsor when appropriate.

**Steering Committee**

The Steering Committee is composed of the Executive Committee and investigators from all participating centers, usually the principal investigators of every center. The Steering Committee will meet periodically to assess the progress, provide scientific input and address policy issues and operational aspects of the protocol. Representatives of the sponsor may attend these meetings as non-voting members.

**Data and Safety Monitoring Board**

An independent Data and Safety Monitoring Board (DSMB) will monitor the accruing safety and outcome data regularly. The DSMB will be composed of Yucheng Chen (independent cardiologist), Ning Guo (independent interventionist), and Guanjian Liu (independent biostatistician). The DSMB operations will be formally separated from the sponsor, the investigators and the Steering/Executive Committee. The DSMB will advise the Chairman of Executive Committee by giving recommendations on trial continuation/discontinuation or aspects of study conduct.

**Clinical Events Committee**

A blinded and independent Clinical Events Committee (CEC) will adjudicate the clinical endpoints. The CEC is composed of three independent interventional cardiologists, Xuebo Liu, Hua Yan, and Xiaofan Wu. The membership will meet periodically and is responsible for reviewing each event as defined in the protocol. Their work is independent and impartial. Such type of review is to ensure accurate counting, assessing, rating of protocol-defined events.

**Core Laboratory for MRI data analysis**

A blinded and independent Core Laboratory will be responsible for MRI analysis. The core laboratory is composed of two independent MRI technicians, Lu Tang and Qiao Deng. They are responsible for viewing and analyzing all image data of patients from West China Hospital and all participating centers. Their work is rigorously kept independent and impartial. Any discrepancy between the analysts will be referred to a senior technician to decide.

**Sup C. Clinical Endpoint definitions**

| **Adverse Clinical Events** | **Definition** |
| --- | --- |
| Cardiac and non-cardiac death | All deaths with a clear cardiovascular (e.g., MI, low-output failure, fatal arrhythmia) or unwitnessed/unknown cause, and procedure-related death (e.g., death related to concomitant treatment), will be classified as cardiac death. Note that deaths caused by vascular cause including cerebrovascular disease, pulmonary embolism, acute aortic syndromes are vascular death, which will not be classified as cardiac death. Any death not covered by the above definitions will be documented as non-cardiac death (e.g., infection, accident, malignancy, or suicide) |
| Non-fatal reinfarction | An acute MI that occurs within 28 days of the incident MI is recognized as re-infarction. Patients with ST-elevation ≥ 1 mm or new pathognomonic Q waves in at least two contiguous leads, particularly with recurrent symptoms or signs of cardiac ischemia should be suspected with re-infarction. An immediate measurement and a second sample obtained 3-6 hours later of cTnT are required. Diagnosis of re-infarction requires a > 20% increase of the cTnT in the second sample. |

MI: myocardial infarction; cTnT: cardiac troponin T

**Sup D. Safety endpoint definitions**

| **Safety Endpoint** | **Definition** |
| --- | --- |
| Worsening renal function | An increase in serum creatinine ≥ 0.5 mg/dL, or a decrease in eGFR ≥ 25% |
| Symptomatic hypotension | clinical symptoms of low cardiac output, such as lightheadedness, weakness, blurred vision, and syncope, with systolic blood pressure ≤90 mm Hg and/or diastolic blood pressure ≤ 60 mm Hg. |
| Hyperkalemia | Potassium levels >5.5 mEq/L |
| Angioedema | The events that resemble angioedema will be carefully evaluated by a separate angioedema adjudication committee to be excluded. |

eGFR: estimated glomerular filtration rate
